# “cAMP Sponge”: A Buffer for Cyclic Adenosine 3′, 5′-Monophosphate

**DOI:** 10.1371/journal.pone.0007649

**Published:** 2009-11-03

**Authors:** Konstantinos Lefkimmiatis, Mary Pat Moyer, Silvana Curci, Aldebaran M. Hofer

**Affiliations:** 1 VA Boston Healthcare System and the Department of Surgery, Brigham and Women's Hospital and Harvard Medical School, West Roxbury, Massachusetts, United States of America; 2 INCELL Corporation LLC, San Antonio, Texas, United States of America; University of California, Berkeley, United States of America

## Abstract

**Background:**

While intracellular buffers are widely used to study calcium signaling, no such tool exists for the other major second messenger, cyclic AMP (cAMP).

**Methods/Principal Findings:**

Here we describe a genetically encoded buffer for cAMP based on the high-affinity cAMP-binding carboxy-terminus of the regulatory subunit RIβ of protein kinase A (PKA). Addition of targeting sequences permitted localization of this fragment to the extra-nuclear compartment, while tagging with mCherry allowed quantification of its expression at the single cell level. This construct (named “cAMP sponge”) was shown to selectively bind cAMP *in vitro*. Its expression significantly suppressed agonist-induced cAMP signals and the downstream activation of PKA within the cytosol as measured by FRET-based sensors in single living cells. Point mutations in the cAMP-binding domains of the construct rendered the chimera unable to bind cAMP *in vitro* or *in situ*. Cyclic AMP sponge was fruitfully applied to examine feedback regulation of gap junction-mediated transfer of cAMP in epithelial cell couplets.

**Conclusions:**

This newest member of the cAMP toolbox has the potential to reveal unique biological functions of cAMP, including insight into the functional significance of compartmentalized signaling events.

## Introduction

Cyclic adenosine 3′, 5′-monophosphate (cAMP) has long been regarded as a “simple” freely diffusible second messenger, well-known for its ability to modulate multiple cellular functions such as motility, secretion, growth, metabolism, and synaptic plasticity[1], [2]. Classically, cAMP signals are initiated by the binding of a specific extracellular ligand to a G-protein-coupled receptor (GPCR) that is linked to a heterotrimeric G-protein containing a Gα_s_ subunit. The ensuing activation of transmembrane adenylyl cyclases results in the production of cAMP. However, the binding of a single ligand to a hormone receptor can set into motion a complex ramifying cascade of signal transduction events that form unpredictable, nonlinear signaling networks[3]. For example, in many cases, a single GPCR is able to interact simultaneously with more than one class of Gα subunit (Gα_i/o_, Gα_q/11_, or Gα_12/13_), generating multiple signals inside the cell. Furthermore, the beta-gamma subunits of heterotrimeric G-proteins, which dissociate following receptor activation, have their own set of biological activities (e.g. modulation of plasma membrane ion channels). Understanding which biological actions are specifically attributable to cAMP amongst all these possible intermediates has presented a longstanding challenge in the signal transduction field[4].

In addition, recent data have revealed an unexpected degree of organizational and spatial complexity in the cAMP signal at the single cell level, showing the existence of localized cAMP-dependent signaling events. For example, soluble isoforms of adenylyl cyclase and localized phosphodiesterases have been linked to the generation of cAMP microdomains, forcing us to re-evaluate the traditional concept that this messenger serves as a straightforward “on-off” switch throughout the bulk cytoplasm[5], [6], [7], [8]. New efforts are now directed at determining whether cAMP microdomains, located for example, within the nucleus, mitochondria, and sub-plasma membrane compartments, are subject to independent and unique modes of regulation. Unfortunately the lack of tools to selectively perturb these subcellular domains has presented a significant obstacle to understanding the potential biological role of localized cAMP signaling events.

The ability to “buffer” cAMP in the cytosol and in specific microdomains might help to resolve these issues. We therefore examined whether it is possible to exploit the high-affinity cAMP-binding portions of the regulatory subunits of protein kinase A (PKA) as a molecular approach for controlling intracellular elevations of cAMP. PKA is the primary effector of the cAMP signal, and consists of two catalytic subunits (PKA-C) bound non-covalently to a dimer of regulatory subunits (PKA-R). Cyclic AMP binding to PKA-R leads to dissociation of the holoenzyme into a PKA-R subunit dimer (with four cAMP molecules bound) and two active C monomers. There are two classes of PKA regulatory subunits (RI and RII) and each of these exist as two subtypes, α and β. The RI subunits have the highest affinity for cAMP and consequently give rise to PKA holoenzymes with lower thresholds of activation as compared to the PKA-RII holoenzymes [2], [9]. The first 100 amino acids (aa) of PKA-RI contain the biologically active domains responsible for homo-dimerization and binding to the PKA-C subunit while the two cAMP binding domains are located in the carboxy terminus [2], [10].

In the present study we describe a targeted high-affinity cAMP buffer based on the carboxy-terminal cAMP-binding fragment of the regulatory subunit RIβ. Over-expression of this “cAMP sponge” was able to buffer agonist-induced cAMP signals as measured at the single cell level and also blocked the downstream activation of PKA. Finally we used this tool to show that cells without the buffer serve as a source of cAMP when coupled via gap junctions to cells harboring the cAMP sponge, and produce extra cAMP to compensate for the extra buffering power provided by the sponge construct.

## Results

### Generation of the cAMP Sponge and of a cAMP-Resistant Mutant Version

**Figure 1:**
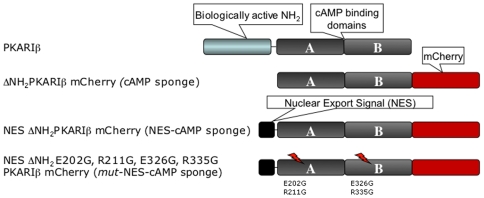
Schematic overview of the strategy used for the generation of cAMP sponge constructs. We cloned the PKA-RIβ C-terminus (AA 133–380), purposely omitting the PKA catalytic inhibitory domain located at N-terminus (AA 90–100). This construct was tagged at its C-terminus with the improved far-red fluorescent protein, mCherry (ΔNH_2_PKARIβ-mCherry). In order to generate a cAMP sponge that was specifically localized to the cytoplasm, we appended the nuclear exclusion signal sequence (*NES*: ALPPLERTLTL) at the N-terminus, generating NESΔNH_2_PKARIβ-mCherry (NES-cAMP sponge). Finally, in order to obtain a cAMP-resistant sponge we mutated the four critical cAMP-binding amino acids in the construct NESΔNH_2_ E202G, R211G, E226G, R335G PKARIβ-mCherry, which we called *mut*-NES-cAMP sponge.

We cloned the PKA-RIβ C-terminus (AA 133–380), purposely omitting the PKA catalytic inhibitory domain located at N-terminus (AA 90–100). This construct binds cAMP with high affinity, but is unable to generate dimers or bind PKA-C [11]. By labeling our chimera with the red fluorescent protein, mCherry (a gift of Roger Tsien[12]) we generated a non-targeted “cAMP sponge” construct. The addition of targeting motifs permitted localization to nuclear, plasma membrane, and cytosolic (i.e. non-nuclear) compartments. We extensively characterized this latter cytosolic construct, bearing the N-terminal nuclear exclusion signal (NES: ALPPLERTLTL). As a control, we also generated a mutant version of this protein unable to bind cAMP called “*mut*-NES-cAMP sponge” in which four point mutations were introduced, two per each of the cAMP binding sites[2] (**Figure 1**).

**Figure 2:**
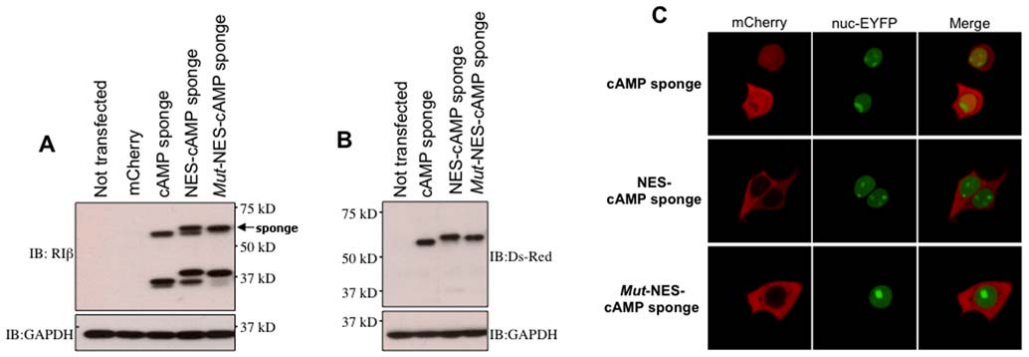
Expression and subcellular localization of cAMP sponge constructs. Western blot analysis using: (**A**) PKA-RIβ specific antibody, and (**B**) Ds-Red antibody that recognizes mCherry. (**C**) Confocal photomicrographs of NCM460 cells co-expressing cAMP “sponges” (mCherry) and a nuclear-targeted EYFP (nuc-EYFP). The chimera named cAMP sponge was present throughout the cell without a specific subcellular localization. The addition of an amino terminus nuclear exclusion signal sequence caused the constructs NES-cAMP sponge and its mutant (*mut*-NES-cAMP sponge) version to be confined within the cytoplasm. Figures are representative of three biological replicates, and the observed localization efficiency was always more that 85% of the cells.

We assessed the expression of our constructs by western blots from total lysates of NCM460[13] cells (a human colonic epithelial cell line) transiently expressing these chimeras. Bands of the expected molecular weights (≈60 kD) were detected using either a PKA-RIβ specific antibody (**Figure 2a**), or one that recognized mCherry (**Figure 2b**). We noted that the PKA-RIβ antibody also reacted with a second set of bands (≈35 kD) likely attributed to extraction-dependent proteolysis of the full-length expressed protein[14]. Confocal imaging of live NCM460 cells expressing the three different cAMP sponge constructs showed similar expression levels as measured by mCherry intensity and the expected subcellular distribution (i.e. non-targeted vs. nuclear exclusion; **Figure 2c**).

### The cAMP Sponge Binds *In Vitro* cAMP While Its Mutant Version Does Not

**Figure 3:**
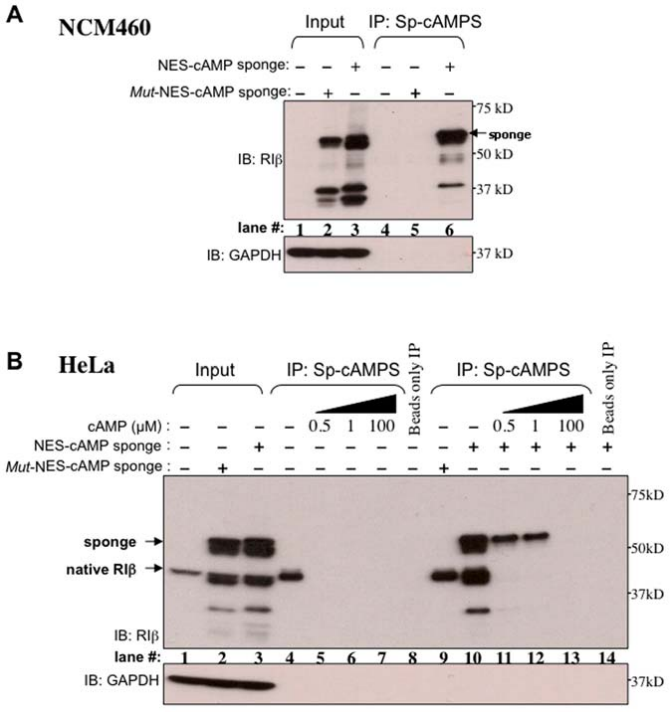
Cyclic AMP sponge is able to bind cAMP in vitro. (A) NCM460 cell lysates immunoprecipitated (IP) using Sp-2-AEA-cAMPS-Agarose beads (Sp-cAMPS): lanes 1–3: input, 4–6: IP, 4: untransfected, 5: *mut*-NES-cAMP sponge, 6: NES-cAMP sponge. (B) cAMP competitive assay, HeLa cell lysates, lanes 1–3: input, 4–14: IP, 4–8: untranfected, 9: *mut*-NES-cAMP sponge, 10–14: NES-cAMP sponge, lanes 8 and 14: beads only. Loading control: Glyceraldehyde-3-phosphate dehydrogenase (GAPDH).

The PKA-RI cAMP binding domains are known to be stable structures that bind cAMP when separated from the rest of the protein[2], [10], [11]. In order to confirm that the ability to bind cAMP was retained in the chimeric sponge proteins, we performed immunoprecipitation experiments using agarose beads coated with a cAMP analog, Sp-2-AEA-cAMPS-Agarose (Biolog) (see **Methods**). We used lysates from NCM460 cells transfected with our sponge constructs, or as controls, untransfected cells. As shown in **figure 3a** the cAMP sponge construct was enriched in the precipitates (lane 6), while as expected, no binding was detected for its mutant version (lane 5) or the untransfected cells (lane 4).

We also tested the cAMP-binding specificity in pull down assays where increasing doses of exogenous cAMP competed with the Sp-2-AEA-cAMPS coating the agarose beads. Both NCM460 and HeLa cells were used because the latter express endogenous PKA-RIβ, making possible the comparison of our RIβ-based chimeras to the endogenous protein. Low concentrations of cAMP (0.5 µM-2.5 µM) drastically reduced the binding of both the endogenous PKA-RIβ, and of cAMP sponge, which was completely abolished at concentrations above 10 µM (**Figure 3b** and supplementary **Figure S1**). In contrast, addition of 1 µM or 5 µM of guanosine 3′, 5′-cyclic monophosphate (cGMP) did not displace our constructs (or the endogenous PKA-RIβ) from the beads (supplementary **Figure S2**). These experiments confirmed that our construct specifically bound cAMP *in vitro* with roughly submicromolar affinity, and that the mutant version lacked this ability.

### cAMP Sponge Attenuates cAMP Signals in Living Cells

**Figure 4:**
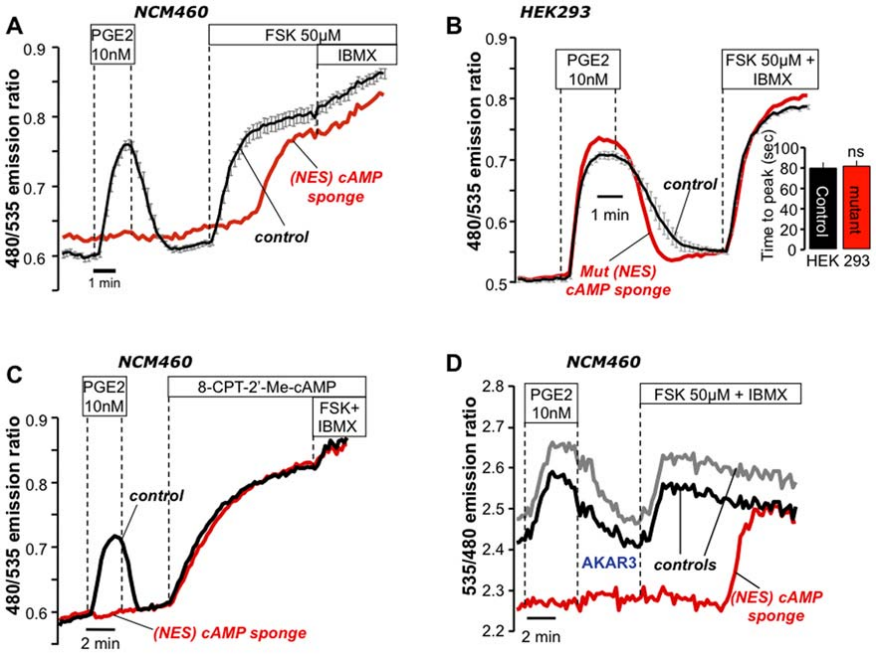
cAMP sponge abolishes agonist-induced cAMP signals and downstream activation of PKA. (A) Experiments in NCM460 cells stably expressing cAMP sensor EpacH30. Cells transiently expressing NES-cAMP sponge (identified by mCherry; red trace) showed significant attenuation of PGE_2_-induced cAMP signals as compared to control cells in same field (black trace; mean ± SEM of 6 cells), typical of 78 controls, 19 cAMP sponge cells in 11 experiments. (B) HEK293 cells expressing *mut*-NES-cAMP sponge (red line) showed no significant differences as compared to control cells (black trace; mean of 4 cells). Inset: time to peak of PGE_2_ response; paired data of 33 controls, 6 *mut*-NES-cAMP sponge 5 experiments, n.s. (C) NCM460 cells treated with the cell permeable EPAC-specific cAMP analog (8-(4-chloro-phenylthio)-2′-*O*-methyladenosine-3′,5′-cyclic monophosphate. (D) NCM460 cells expressing AKAR3 plus NES-cAMP sponge (red trace) showed no PKA activation due to PGE_2_ challenge, in contrast to controls expressing AKAR3 alone in the same field (black and gray traces).

We used the FRET-based cAMP sensor “Epac H30” which is built around the native cAMP-binding protein Epac in order to assess the effectiveness of our buffers at the single cell level[15], [16]. These experiments were conducted in NCM460 and HEK293 cell lines stably expressing the Epac H30 sensor (see **Methods**). These cells were transiently transfected with cAMP sponge, and cAMP responses of single, isolated sponge-transfected cells (identified by mCherry fluorescence) were directly compared to neighboring control cells in the same microscope field. As shown in **Figure 4a** all controls responded to prostaglandin E_2_ (PGE_2_, black line), while the sponge-expressing cells (red line) typically gave no response. Of 19 isolated sponge-expressing cells examined in 11 experiments, there were four cells that did respond weakly to PGE_2_, but with a >3-fold time delay as compared to the controls (supplementary **Figure S3**). Supra-maximal doses of forskolin (FSK; a nonspecific adenylyl cyclase activator) combined with the general phosphodiesterase inhibitor isobutylmethylxanthine (IBMX), caused the cAMP sponge to eventually become saturated, yielding a response similar to isolated control cells. Similar experiments were performed using HEK293 cells (supplementary **Figure S4**). In contrast, isolated HEK293 cells (**Figure 4b**; typical of 33 control, 6 cAMP sponge cells in 5 experiments), and NCM460 cells (supplementary **Figure S5**; 74 controls, 10 cAMP sponge cells in 6 experiments) expressing the mutant cAMP sponge showed no significant differences in the amplitude or timing of the response as compared to the controls.

As a further control to confirm that the Epac H30 FRET sensor was competent to respond to cAMP in the sponge-expressing cells, we used a cell-permeable Epac-specific cAMP analog, 8CPT-2Me-cAMP (8-(4-chloro-phenylthio)-2′-*O*-methyladenosine-3′,5′-cyclic monophosphate)[17]. This compound binds to native Epac and the Epac H30 sensor, but not to the PKA-RIβ. We therefore expected that the PKA-RIβ-based cAMP sponge would not recognize 8CPT-2Me-cAMP. In fact, no differences between control and sponge-expressing cells were observed when the cells were treated with the Epac-specific analog, whereas the response to an elevation in native cAMP was clearly affected (**Figure 4c**; 41 controls, 10 cAMP sponge cells in 5 experiments).

### Overexpression of cAMP Sponge Blocks the Activation of the Main cAMP Effector, PKA

We next examined whether cAMP sponge, by damping free [cAMP], would also attenuate the activation of PKA during agonist stimulation. For this purpose, we used two genetically encoded sensors, AKAR2 and AKAR3 (gifts of Roger Tsien and Jin Zhang[18], [19]), that report phosphorylation by PKA *via* a change in FRET. These sensors do not bind cAMP directly. As shown in **Figure 4d**, NCM460 cells transfected with AKAR3 alone responded normally to PGE_2_ stimulation, while neighboring cells co-expressing AKAR3 and the cAMP sponge showed no significant FRET response, indicating a lack of PKA activity. As expected FSK plus IBMX eventually saturated the buffer, restoring the PKA activity in the sponge-expressing cells (typical of 10 controls, 10 sponge cells in 6 experiments). These data provide independent confirmation that cAMP sponge can effectively dampen cAMP signaling, measured as a loss of activation of the major downstream target of the cAMP signal, PKA.

### cAMP Sponge-Expressing Cells Serve as a Sink for cAMP Generated in Neighboring Cells Connected via Gap Junctions

**Figure 5:**
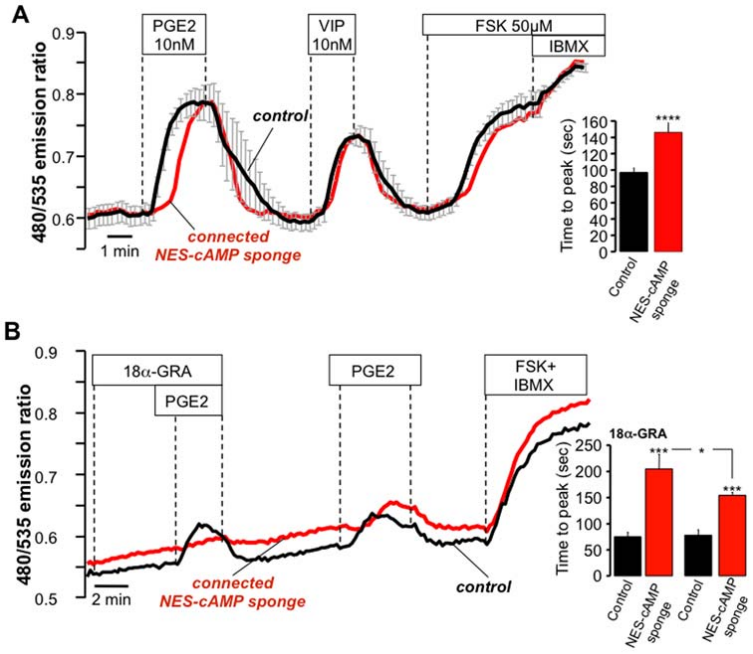
Transfer of cAMP from control cells to connected buffer expressing cells through gap junctions. (a) NCM460 cells expressing NES-cAMP sponge (red line) connected to control cells (black line; mean of 4 cells), showed a small delay (time to peak) in the PGE_2_ response (inset: mean ± s.e.m. of 49 controls, 11 NES-cAMP sponge in 9 experiments). (b) Pre-incubation with the gap-junction inhibitor 18α-glycyrrhetinic acid (18α*-GRA*) significantly increased the time to peak of the buffer expressing cells. Inset: summary of 46 controls, 10 cAMP sponge cells in 6 experiments (* p<0.05; *** p<0.001; **** p<0.0001). See also supplementary Movie S1.

The coordinated physiological activity of many tissues relies on cell-to-cell transfer of metabolites, electrical signals, and second messengers (including cAMP) via gap junctions[20], [21]. Imaging studies using FRET-based sensors have shown that cAMP levels in individual cells follow those of the surrounding cells due to diffusion through these junctions[20], [21]. We questioned how the presence of the cAMP buffer would affect the agonist-stimulated cAMP signal when buffer-expressing cells were physically connected to non-transfected controls. To this aim we sought out couplets of NCM460 cells in the microscope field in which one of the cells expressed the cAMP sponge construct and the other did not (control cell). Time-lapse images of the Epac H30 FRET ratio during agonist stimulation suggested that the control cells were acting as a source of cAMP, while the connected buffer-expressing cells served as a sink for the second messenger (supplementary **Movie S1**). As shown in **Figure 5a**, under these conditions there was a significant delay in the PGE_2_ response, averaging ∼50 seconds in the cAMP sponge cells, compared to untransfected controls (49 controls, 11 mCherry cells, 9 experiments p<0.0005).

Pretreatment with the reversible gap junction blocker 18α-glycyrrhetinic acid[22] inhibited the cell-to-cell transfer of cAMP, and this was translated into a doubling of the delay in the agonist response observed in cAMP sponge-expressing cells. When 18α-glycyrrhetinic acid was rinsed away, the cAMP transfer from controls to buffer expressing cells was rescued, with a significantly shorter delay in the response (**Figure 5b**; 46 controls, 10 cAMP sponge cells in 6 experiments). It is noteworthy that the amplitude and time course of the cAMP response in the control cell was the same independent of whether cAMP was permitted to diffuse into the buffer-expressing cell via the 18α-glycyrrhetinic acid-dependent pathway. This would suggest that second messenger produced in one cell is able to compensate for a lagging cell, otherwise the cAMP response in the control cell would have been larger in the presence of the gap junction inhibitor.

## Discussion

The introduction of cell-permeant calcium chelators such as BAPTA-AM (1,2-bis(o-aminophenoxy)ethane-N,N,N',N'-tetraacetic acid, acetoxymethyl ester) by Roger Tsien in the early 1980's [23], [24] revolutionized the study of Ca^2+^ signaling. This high affinity Ca^2+^ chelator can be loaded non-invasively into living cells, and used to rapidly clamp [Ca^2+^] in the cytosol to resting levels during agonist activation. This invaluable tool permitted investigators to dissect out the relative importance of the Ca^2+^ spike in complex systems involving concurrent activation of multiple signaling pathways. Low-affinity Ca^2+^ buffers such as N,N,N',N'-tetrakis (2-pyridylmethyl)ethylene diamine or TPEN, used previously to clamp [Ca^2+^] within endoplasmic reticulum Ca^2+^ stores[25], have also proven useful for reversibly manipulating free [Ca^2+^] within subcellular compartments.

Uchiyama and colleagues extended this concept by generating the first genetically encoded buffer for inositol 1,4,5-trisphosphate (IP_3_). This construct (named “IP_3_ sponge”)[26] was based on a hyperaffinity IP_3_ binding fragment derived from the type I IP_3_ receptor. Surprisingly, to date no such tool has been described for the ubiquitous intracellular second messenger, cAMP, prompting us to develop the “cAMP sponge” constructs described here.

In order to be effective as a buffering molecule, the affinity of a cAMP sponge should be somewhat less than the resting free levels of cAMP (maintained by the constitutive action of PDEs), otherwise the buffer molecule would be saturated prior to stimulation. It should be able, however, to compete with endogenous effectors of the cAMP signal, e.g. Epac, PKA, and cyclic nucleotide-gated channels. Recent work by Døskeland and colleagues has shown that the cAMP affinity of the PKA holoenzyme and Epac are similar (about 2.9 µM), but that the isolated RIα has about three orders of magnitude higher affinity for cAMP (≈0.9 nM) [9]. While native PKA-RI subunits could potentially act as endogenous high affinity soluble cAMP buffers, free regulatory subunits are rarely found in the living cell because their expression levels are tightly controlled in a 1∶1 ratio with those of PKA-C [27], [28].

In our study, we constructed a cAMP buffer based on the tandem cAMP-binding domains of the isolated PKA-RIβ. This truncated form of RIβ is unable to bind the PKA catalytic subunit or to dimerize with itself. Our construct was shown to bind cAMP *in vitro* with roughly submicromolar affinity, and was insensitive to cGMP. The fragment was tagged with a fluorescent protein variant, mCherry, which is spectrally compatible with the CFP and YFP of FRET-based sensors for cAMP. This permitted correlation of the concentration of the expressed buffer (a function of mCherry fluorescence intensity) with its actions on cAMP signaling as measured by an Epac- and FRET-based cAMP sensor [16] in single cells. We were also successful in targeting our construct to the cytoplasm using a classic nuclear exclusion signal, proving its suitability for sub-cellular localization. Finally, the introduction of four point mutations led to the generation of a double mutant version unable to bind cAMP, which has provided an optimal control.

We validated cAMP sponge at the single cell level using a FRET-based imaging approach and demonstrated that it was able to block agonist-induced cAMP elevations (EPAC H30, figure 4a–c) and the downstream PKA activation (AKAR3, Figure 4d). In contrast, in experiments performed using the mutant version of our sponge, no significant effect on the cAMP signal was measured.

To illustrate a practical application for this tool, we probed the effect of the cAMP buffer on intercellular transfer of cAMP *via* gap junctions by analyzing couplets of NCM460 colonic epithelial cells in which only one of the two cells expressed the cAMP sponge. Our data suggest that during agonist challenge, control cells produced extra cAMP that diffused into neighboring cells until the additional buffering capacity of the expressed sponge construct was overwhelmed, leading to a detectable elevation of free cAMP. These data bring to light the intriguing possibility that some type of feedback regulation allows cAMP to control its own permeation through gap junctions. It is known, for example, that PKA can phosphorylate certain connexin proteins (the elemental components of the gap junction), leading to alterations in gap junction permeability[29], [30]. This could potentially provide a mechanism that allows cells to “sense” the lack of free second messenger in one cell, and compensate by increasing the sharing of cAMP *via* this pathway. It is perhaps relevant that agonist-activated cAMP signals of individual NCM460 cells within coupled cell clusters were highly homogenous with respect to amplitude and time course under control conditions, but were strikingly heterogeneous in the presence of gap junction inhibitors (KL and AMH, unpublished observations). These observations would be consistent with a role for gap junction-mediated sharing of cAMP in “normalizing” the signal across epithelial sheets.

The second messenger concept, as proposed many decades ago, originally portrayed global, uniform elevations of Ca^2+^ and cAMP as simple on/off switches for controlling cell function. Sophisticated tools for monitoring and manipulating the Ca^2+^ signal (including Ca^2+^ buffers) showed, however, the functional importance of highly localized, elementary Ca^2+^ signaling events (Ca^2+^ sparks, puffs, and blips). Does something akin to a “cAMP spark” also exist, and does it encode unique information? While recent data have pointed to the existence of privileged cAMP signaling microdomains which have the potential to differentially control cellular functions[6], the development of tools to selectively perturb these signals has not kept pace with this rapidly expanding area of investigation. The possibility to clamp [cAMP] in highly localized subcellular microdomains using targeted “cAMP sponge” constructs described here should prove useful for interrogating this previously inaccessible aspect of the cAMP signal transduction process.

## Materials and Methods

### Reagents

Guanosine 3′, 5′-cyclic monophosphate (cGMP) and 8CPT-2Me-cAMP (8-(4-chloro-phenylthio)-2′-*O*-methyladenosine-3′, 5′-cyclic monophosphate) were obtained from Calbiochem (San Diego, CA). 2- (2- Aminoethylamino) adenosine- 3′, 5′- cyclic monophosphorothioate, Sp- isomer, (Sp-2-AEA-cAMPS-Agarose) was obtained from Biolog (Biolog, Hayward CA). All restriction enzymes were purchased from New England Biolabs (Ipswich, MA). Primers were custom made by Invitrogen (Carlsbad, CA). All other reagents were from Sigma (St Louis, MO) unless otherwise noted.

### Cell Culture and Transfection

HeLa and human embryonic kidney (HEK293) cells (ATCC) were grown in Dulbecco's modified Eagle's medium (DMEM) supplemented with 10% fetal calf serum. NCM460 cells were obtained by a licensing agreement from INCELL Corporation, LLC, (San Antonio TX), and grown in M3:10 medium (INCELL) according to the supplier's recommendations. HEK293 and NCM460 cell lines stably expressing the cAMP sensor Epac H30 were generated by repeated rounds of sorting using FACS (fluorescence activated cell sorter; Beth Israel Deaconess Medical Center Flow Cytometry Core, Boston MA). All constructs were transfected using Effectene transfection reagent (Qiagen, Valencia, CA) according to the manufacturer.

### Expression Plasmids and Mutagenesis

To generate the cAMP sponge constructs, we used specific primers in polymerase chain reactions (PCR) to amplify amino acids 132 to 381 of the human PKA regulatory subunit Iβ (PKA-RIβ; Origene clone TC 124688, NM_002735). We used Pfu Ultra High-fidelity DNA Polymerase (Stratagene, La Jolla, CA), with the following primers carrying a 5′ Bam HI restriction enzyme sites (underlined): Primer Forward 5′-GTGGATCCAATCTCCAAGAACGTGCTCTTC-3′, primer Reverse 5′-GTGGATCCGCGACGGTGAGGGAGATGAAGCT-3′. The PCR product after digestion was subcloned in frame with mCherry into the vector pcDNA3.

The Nuclear Export Signal (NES: ALPPLERTLTL)[10] was added at the N-terminus to obtain cytoplasmic (non-nuclear) localization of our construct. Two rounds of site directed mutagenesis (Quickchange XL; Stratagene La Jolla, CA) generated the four point mutations (E202G, R211G, E326G, R335G) that altered the critical cAMP binding residues for both binding sites. All constructs were sequenced (Dana Farber DNA Resource Core, Boston MA).

### Confocal Imaging

NCM460 cells were seeded on glass coverslips and after 24 hours were co-transfected with equal amounts of cAMP sponge constructs and an enhanced Yellow Fluorescent Protein targeted to the nucleus (nuc-EYFP). Twenty-four to forty-eight hours after transfection, the coverslips were mounted in a home-built flow through perfusion chamber. Cells bathed in HEPES-buffered Ringer's solution were imaged under a 60X oil immersion objective on a Nikon Confocal Microscope C1. Images were collected using the EZ-C1 software (Nikon).

### Western Blot and Immunoprecipitation

HeLa or NCM460 cells were transfected with equal amounts of (NES) cAMP sponge or mutant (NES) cAMP sponge. Twenty-four hours after transfection, cells were lysed using RIPA buffer (Sigma) complemented with protease inhibitors cocktail (Sigma) or SolObuffer (FabGennix Inc., Frisco, TX) complemented with protease inhibitor cocktail (FabGennix Inc.). Cell lysates were sonicated and insoluble material was removed by centrifugation at 14,000 g for 10 min at 4°C. For immunoprecipitation experiments 100 µg–200 µg of NCM460 or HeLa lysates were incubated for 2 h at 4° with 25 µl of agarose beads coated with the PDE-resistant cAMP analog 2- (2- Aminoethylamino) adenosine- 3′, 5′- cyclic monophosphorothioate, Sp-isomer, (Sp-2-AEA-cAMPS-Agarose; from Biolog, Hayward CA). Beads were washed three times with ice-cold PBS and the proteins were released with 25 µl–35 µl of 2x SDS-loading buffer. Equal amount of immunoprecipitated proteins or total cell lysates were resolved on 5%–20% tris-glycine SDS/PAGE gels and electroblotted onto PVDF membranes (Hybond-P, Amersham Biosciences, Piscataway, NJ). After transfer PVDF membranes were blocked for 1 h at room temperature in 5% milk with Tris-buffered saline/Tween 20 (TBST; 10 mM Tris HCl, pH 8.0/150 mM NaCl/0.1% Tween 20). Next the membranes were incubated for 2 h at room temperature with an anti-PKA-RIβ antibody (c19, Santa Cruz Biotechnology Inc., Santa Cruz, CA) or an anti-DS-red antibody that also recognizes mCherry (Clontech, Mountain View, CA). Both primary antibodies were diluted 1∶1000 in 5% milk-TBST. After four washes with TBST, membranes were incubated at room temperature for 1 h with a peroxidase-conjugated secondary antibody (1∶2000; Santa Cruz). Peroxidase activity was detected with enhanced chemiluminescence (ECL advance western blotting detection kit, Amersham Biosciences). Glyceraldehyde-3-phosphate dehydrogenase (GAPDH) antibody (1∶2000; Santa Cruz) was used as a loading control for the total cell lysates and to detect protein contamination for the immunoprecipitated proteins.

### Ratio Imaging

Real-time FRET imaging experiments were performed using a fluorescence imaging system built around a Nikon TE200 microscope as previously described[31]. Metafluor software (Molecular Devices, Downingtown, PA) was used to control filter wheels (Sutter Instruments, Novato, CA) placed in the excitation and emission path, and to acquire ratio data. Cells were seeded on glass coverslips and were transfected 24 hours later. The following day, we mounted the coverslips in a home-built flow through perfusion chamber, and imaged the cells using a 40X oil immersion objective. Cells were bathed in HEPES-buffered Ringer's solution containing (in mM): 125 NaCl, 25 HEPES, 10 Glucose, 5 K_2_HPO_4_, 1 MgSO_4_ and 1 CaCl_2_, pH = 7.40. The 485 nm/535 nm FRET emission ratios from the Epac-based cAMP sensor (440 nm excitation) were acquired every 10 seconds. PKA phosphorylation activity was expressed as the 535 nm/485 nm FRET emission ratios of AKAR2 or AKAR3 (440 nm excitation). The fluorescence of mCherry (excitation 585 nm, emission 610 nm) did not interfere with either of these measurements as previously reported[32].

## Supporting Information

Figure S1 Cyclic AMP competitive assay. Lysates from NCM460 cells transfected with NES-cAMP sponge, its mutant variant, and untransfected cells were immunoprecipitated using Sp-2-AEA-cAMPS-Agarose (Sp-cAMPS) with increasing concentrations of exogenous cAMP. Lanes 1–7: IP supernatants; lane 8: IP untransfected; lane 9: mut-NES-cAMP sponge; lanes 10–14: NES-cAMP sponge lane 14: beads only. Glyceraldehyde-3-phosphate dehydrogenase (GAPDH) was used to assess protein loading and contamination.
(3.00 MB TIF)

Figure S2 Cyclic GMP competitive assay. HeLa total cell lysates immunoprecipitated (IP) using Sp-2-AEA-cAMPS-Agarose beads (Sp-cAMPS) in the presence of increasing concentrations of cGMP: lanes 1–6: supernatants, 7–12: IP, lane 7: untransfected, 8: mut-NES-cAMP sponge, 9–12: NES-cAMP sponge. Addition of 1–5 µM of exogenous cGMP did not affect the binding of the buffer or the endogenous RIβ to Sp-cAMP, when the cGMP concentration was increased to 100 µM the binding of both (RIβ and buffer) was affected.
(3.00 MB TIF)

Figure S3 Delayed cAMP response in buffer-expressing NCM460 cells. In a subset of NCM460 cells stably expressing cAMP sensor EpacH30 transfected with (NES) cAMP sponge (red trace) there was a small response but this occurred with a 3 fold delay in the time to peak as compared to controls in same field (black trace). Typical of 4 (NES) cAMP sponge cells out of 19 in 11 experiments. Upon addition of the cell-permeable EPAC-specific cAMP analog 8-CPT-2′ Me-cAMP, (NES) cAMP sponge-expressing cells responded similarly to the controls.
(3.00 MB TIF)

Figure S4 Expression of cAMP sponge blocks agonist-induced cAMP signals in HEK293 cells. Isolated NES-cAMP sponge-expressing cells (identified by mCherry; red trace) showed no response to PGE2 compared to the untransfected controls (black trace; mean of 6 cells) on the same coverslip. On the other hand cells expressing the buffer that were connected to control cells (red trace) showed a significant delay of the response but eventually responded, indicating the saturation of the buffer. A combination of forskolin (FSK 50 µM) and IBMX (1 mM) saturated the buffer, producing responses similar to the controls (representative data of 28 controls, 8 cAMP sponge in 5 experiments).
(3.00 MB TIF)

Figure S5 The mutant cAMP sponge does not influence the kinetics of PGE2-induced responses. Bar graph indicating PGE2 responses of NCM460 cells expressing mutant (NES) cAMP sponge and controls in the same field. No significant difference was detected (74 controls, 10 cAMP sponge cells in 6 experiments) between the two groups.
(3.00 MB TIF)

Movie S1 Vectorial transfer of cAMP between connected cells. NCM460 cells stably expressing the EpacH30 cAMP sensor were transiently transfected with cAMP sponge. Here the microscope field contained an isolated control cell (on the left) and a couplet of cells in which only one cell expressed the sponge construct (on the right). The image sequence shows a significant delay in the PGE2 response of the coupled sponge-expressing cell compared to the two controls, with an apparent transfer of cAMP from the control (top cell in couplet) to the cell expressing the cAMP sponge (bottom). Time-lapse images represent pseudo-color of EpacH30 480 nm/535 nm FRET emission ratio, one frame every 10 seconds, total experiment duration 5.5 minutes.
(1.40 MB AVI)
