# Supplementary figures and images for: “cAMP Sponge”: A Buffer for Cyclic Adenosine 3′, 5′-Monophosphate
Source: PLoS One. 2009 Nov 3;4(11):e7649. doi: 10.1371/journal.pone.0007649 (PMC2766031; doi:10.1371/journal.pone.0007649)

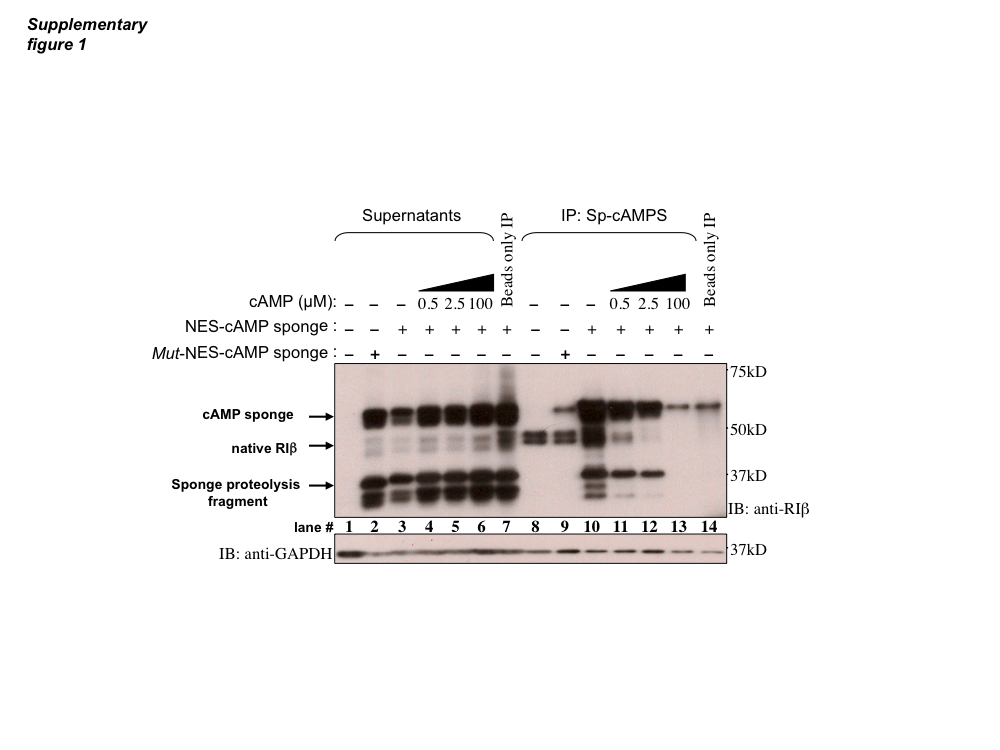

Supplement: Figure S1 — Cyclic AMP competitive assay. Lysates from NCM460 cells transfected with NES-cAMP sponge, its mutant variant, and untransfected cells were immunoprecipitated using Sp-2-AEA-cAMPS-Agarose (Sp-cAMPS) with increasing concentrations of exogenous cAMP. Lanes 1–7: IP supernatants; lane 8: IP untransfected; lane 9: mut-NES-cAMP sponge; lanes 10–14: NES-cAMP sponge lane 14: beads only. Glyceraldehyde-3-phosphate dehydrogenase (GAPDH) was used to assess protein loading and contamination. (3.00 MB TIF) [file pone.0007649.s001.tif]

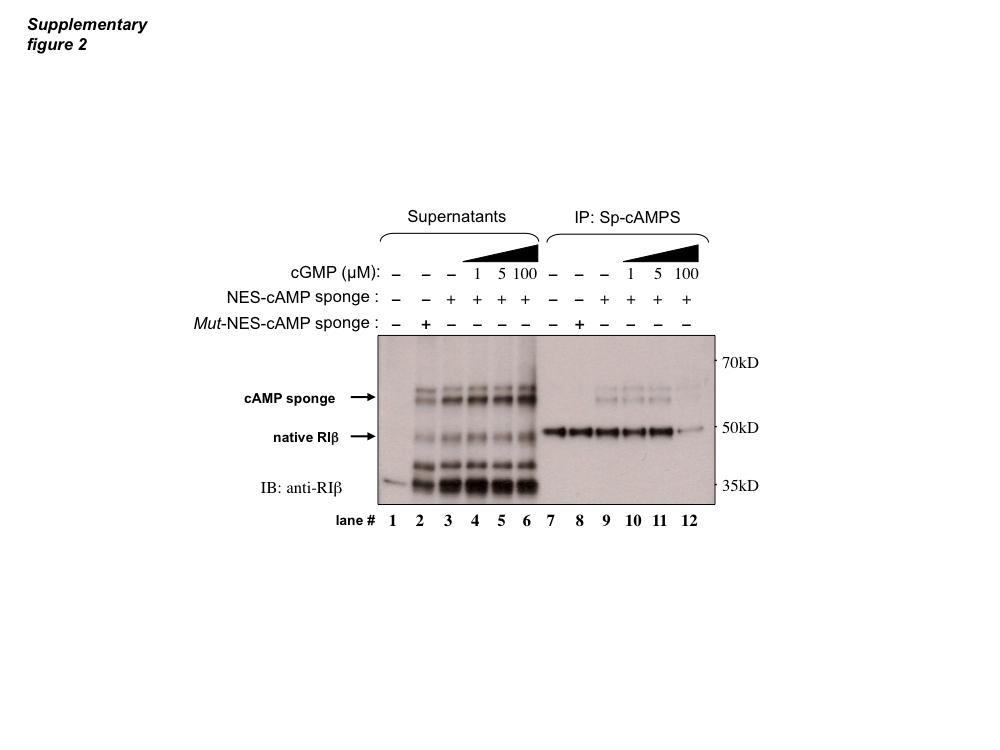

Supplement: Figure S2 — Cyclic GMP competitive assay. HeLa total cell lysates immunoprecipitated (IP) using Sp-2-AEA-cAMPS-Agarose beads (Sp-cAMPS) in the presence of increasing concentrations of cGMP: lanes 1–6: supernatants, 7–12: IP, lane 7: untransfected, 8: mut-NES-cAMP sponge, 9–12: NES-cAMP sponge. Addition of 1–5 µM of exogenous cGMP did not affect the binding of the buffer or the endogenous RIβ to Sp-cAMP, when the cGMP concentration was increased to 100 µM the binding of both (RIβ and buffer) was affected. (3.00 MB TIF) [file pone.0007649.s002.tif]

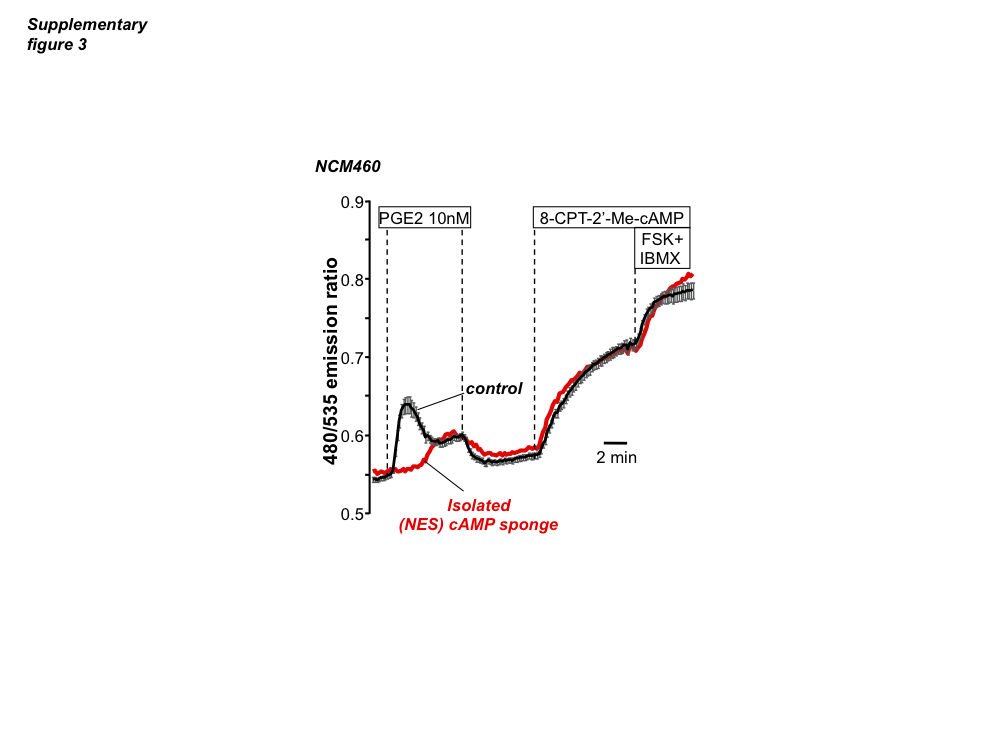

Supplement: Figure S3 — Delayed cAMP response in buffer-expressing NCM460 cells. In a subset of NCM460 cells stably expressing cAMP sensor EpacH30 transfected with (NES) cAMP sponge (red trace) there was a small response but this occurred with a 3 fold delay in the time to peak as compared to controls in same field (black trace). Typical of 4 (NES) cAMP sponge cells out of 19 in 11 experiments. Upon addition of the cell-permeable EPAC-specific cAMP analog 8-CPT-2′ Me-cAMP, (NES) cAMP sponge-expressing cells responded similarly to the controls. (3.00 MB TIF) [file pone.0007649.s003.tif]

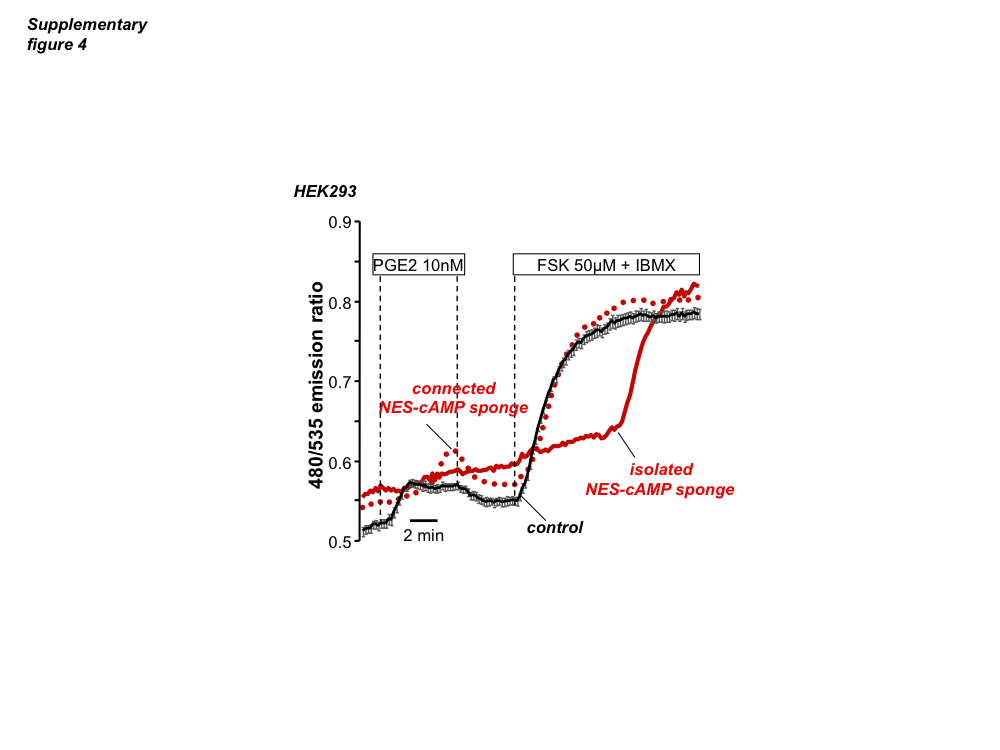

Supplement: Figure S4 — Expression of cAMP sponge blocks agonist-induced cAMP signals in HEK293 cells. Isolated NES-cAMP sponge-expressing cells (identified by mCherry; red trace) showed no response to PGE2 compared to the untransfected controls (black trace; mean of 6 cells) on the same coverslip. On the other hand cells expressing the buffer that were connected to control cells (red trace) showed a significant delay of the response but eventually responded, indicating the saturation of the buffer. A combination of forskolin (FSK 50 µM) and IBMX (1 mM) saturated the buffer, producing responses similar to the controls (representative data of 28 controls, 8 cAMP sponge in 5 experiments). (3.00 MB TIF) [file pone.0007649.s004.tif]

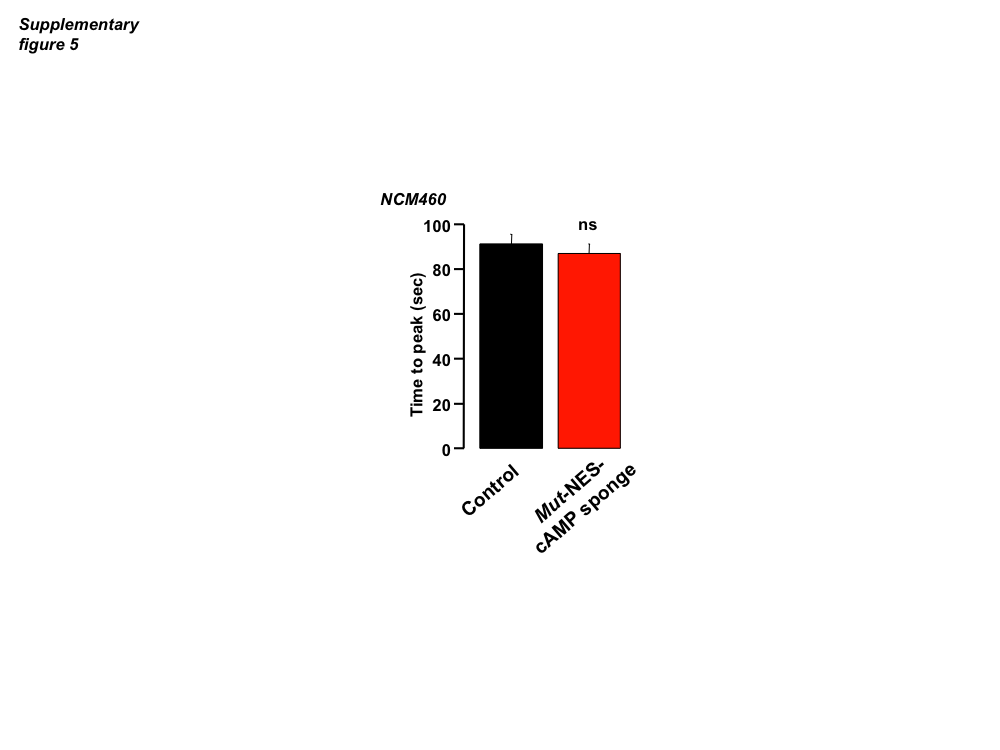

Supplement: Figure S5 — The mutant cAMP sponge does not influence the kinetics of PGE2-induced responses. Bar graph indicating PGE2 responses of NCM460 cells expressing mutant (NES) cAMP sponge and controls in the same field. No significant difference was detected (74 controls, 10 cAMP sponge cells in 6 experiments) between the two groups. (3.00 MB TIF) [file pone.0007649.s005.tif]
